# Living with and beyond cancer with comorbid illness: a qualitative systematic review and evidence synthesis

**DOI:** 10.1007/s11764-019-0734-z

**Published:** 2019-01-26

**Authors:** Debbie Cavers, Liset Habets, Sarah Cunningham-Burley, Eila Watson, Elspeth Banks, Christine Campbell

**Affiliations:** 1 Usher Institute, Medical School, University of Edinburgh, Teviot Place, Edinburgh, EH8 9AG UK; 2 Leiden University Medical Center, University of Leiden, Albinusdreef 2, 2333 Leiden, ZA Netherlands; 3 Medical and Family Sociology, Usher Institute, Medical School, University of Edinburgh, Teviot Place, Edinburgh, EH8 9AG UK; 4 Supportive Cancer Care, Faculty of Health and Life Sciences, Oxford Brookes University, Jack Straws Lane, Marston, Oxford, OX3 0FL UK; 5 1 Carnwath Lane, Carluke, South Lanarkshire ML8 4QU UK

**Keywords:** Comorbidity, Systematic review, Qualitative, Supportive care, Cancer experience, Survivorship care

## Abstract

**Purpose:**

To identify the qualitative evidence on the experience of cancer and comorbid illness from the perspective of patients, carers and health care professionals to identify psycho-social support needs, experience of health care, and to highlight areas where more research is needed.

**Methods:**

A qualitative systematic review following PRISMA guidance. Relevant research databases were searched using an exhaustive list of search terms. Two reviewers independently screened titles and abstracts and discussed variations. Included articles were subject to quality appraisal before data extraction of article characteristics and findings. Thomas and Harden’s thematic synthesis of extracted findings was undertaken.

**Results:**

Thirty-one articles were included in the review, covering a range of cancer types and comorbid conditions; with varying time since cancer diagnosis and apparent severity of disease for both cancer and other conditions. The majority of studies were published after 2010 and in high income countries. Few studies focused exclusively on the experience of living with comorbid conditions alongside cancer; such that evidence was limited. Key themes identified included the interaction between cancer and comorbid conditions, symptom experience, illness identities and ageing, self-management and the role of primary and secondary care.

**Conclusions:**

In addition to a better understanding of the complex experience of cancer and comorbidity, the review will combine with research prioritisation work with consumers to inform an interview study with the defined patient group.

**Implications for Cancer Survivors:**

Expanding this evidence base will help to illuminate developing models of cancer patient-centred follow-up care for the large proportion of patients with comorbid conditions.

**Electronic supplementary material:**

The online version of this article (10.1007/s11764-019-0734-z) contains supplementary material, which is available to authorized users.

## Introduction

Macmillan Cancer Support estimates that there are now 2.5 million people living with and beyond cancer in the UK, and this figure is expected to rise to 4 million by 2030 [1], explained, in part, by the ageing population, and improved screening, earlier diagnosis and better treatments. Deborah Boyle insightfully described the changing ‘cancer patient mosaic’, and the reality is that most people living beyond cancer are living with additional comorbid illness [2]. As many as 78% of cancer patients report at least one other condition, and such multi-morbidity also increases with age [3, 4]. A recent study suggested that cancer survivors had an average of five comorbid conditions, with some of these developing after cancer diagnosis [5]. Commonly reported comorbid conditions among cancer survivors included heart- and lung-related illnesses, ear and eye problems, hypertension, arthritis or rheumatism and depression and anxiety [5, 6]. Prevalence and comorbidity type varied according to time since diagnosis and cancer type, as well as associations with ethnicity, marital status, weight and physical activity [5]. Evidence suggests that the presence of comorbid conditions among those diagnosed with cancer is associated with poorer survival and quality of life, due to increased symptom burden, being less likely to receive treatment with curative intent and these patients are often excluded from clinical trials [7].

Psychosocial support for cancer survivors has become an increasing priority on the policy agenda over the last 10 years, as evidenced by a recent Lancet Oncology paper series devoted to cancer survivorship [8]. In the UK, this policy priority was set out in the Cancer Reform Strategy, which in turn led to the development of the National Cancer Survivorship Initiative in England and Wales [9, 10], and forms part of the remit for Scotland’s Better Cancer Care [11].

Follow-up care and psychosocial support for survivors of cancer presents challenges to health care services [12] and has implications for the role of primary, secondary and community care as new models of follow-up are recommended [13, 14]. This picture becomes more complex in the presence of comorbidities, with implications for the coordination of quality care and support [15, 16]. While valuable research has been conducted to understand the experience of living with and beyond cancer for the patient [10, 17], and their relatives [18–22], less is known about the impact of additional chronic illness. Research exploring the support needs of people living with multiple complex conditions, including cancer, therefore needs to be identified [23]. Service development and provision would also benefit from insights in this area, in order to highlight areas for further research [17, 24].

### Aims and research questions

Understanding the challenges experienced by people living after a cancer diagnosis with other chronic conditions such as COPD, diabetes or mental ill health, can give new insights into patient-centred models of care. A systematic review approach provides a comprehensive process to explore the current evidence base and define a research agenda building on prior knowledge [25]. This systematic review aimed to identify and synthesise qualitative evidence on the experience of living with and beyond cancer with one or more additional long-term illnesses. Insights from the review will inform further research in this area. The review seeks to answer the following questions:

- What qualitative evidence is available that explores the *experience* of living with both cancer and one or more comorbidities from patient, carer and provider perspectives?
- What are the psychosocial support needs of people living with cancer and one or more comorbidity as identified in the literature?
- What are patient, carer and provider experiences of service provision for cancer and comorbid conditions reported in the literature and what research priorities can be derived from the available evidence?

## Methods

The review methods are detailed in full in a published protocol [26]. The methods are summarised below. The review protocol is registered on the International Prospective Register of Systematic Reviews (PROSPERO) database (registration number: CRD42016041796).

### Design and ethics

A review of qualitative papers was chosen to fit with the aim of exploring people’s experiences, using a person-centred approach to highlight context and generate meaningful and relevant findings [27]. The review used methods of qualitative synthesis to combine, integrate and interpret, where possible, the evidence from the included papers [27, 28]. The review aimed to move beyond the aggregation of available data to provide further interpretive insights into living with complex illness and define where future research can add to what is known [28].

The review method follows the PRISMA statement guidance for conducting a systematic review [29].

This review was exempt from NHS and internal University of Edinburgh Usher Institute for Population Health Sciences and Informatics Ethics Review Committee because no primary data was used.

### Eligibility criteria

Articles were included if they were published in English between January 2000–January 2017 to capture the current survivorship agenda while allowing for a broad view of issues as they have developed. Eligible articles included wholly qualitative studies and the qualitative component of mixed methods studies (including unpublished literature) addressing the topic of psychosocial dimensions of living with cancer and comorbid illness (diagnosed before or after the cancer diagnosis but not directly caused by the cancer), including issues of support and experience of service provision from diagnosis to end of life. Adult patients (18 years or over), informal carer and health care professional perspectives were included.

Any cancer type was included along with any comorbidity (as defined by the ISD Scotland report [30]) and listed in Barnett et al.’s paper mapping the epidemiology of multi-morbidity [4], listed in Online Resource 1. Long-term side effects of cancer treatment and second primary cancers were not included. See Online Resource 2 for a summary of inclusion and exclusion criteria.

### Dimensions of interest

The review includes physical, social, emotional, psychological and spiritual dimensions of experience of cancer and comorbidity. Full details of the review topic can be found in the study protocol [26].

### Information sources

Literature was identified using a variety of methods, including database searching, citation and snowball searching, known expert consultation via email and related articles searches in PubMed and Google scholar. The databases consulted were Medline, Embase, CINAHL, PsycINFO, ASSIA, Sociological Abstracts, Web of Science, SCOPUS and, for grey literature, OpenGrey and ProQuest Dissertations and Theses Global.

### Search strategy

A broad search strategy was developed to reflect the exploratory nature of the review (see Online Resource 3 for an example search strategy for Medline, adapted to each database using free text, MeSH and subject headings where possible for maximum sensitivity and specificity).

### Data collection and analysis

#### Study records and screening

Identified records were imported into EndNoteX7 and managed in subsequent databases to track the number of records at each stage of the screening process. Two reviewers (DC and LH) screened articles in three stages: title, abstract and full text. A third reviewer (CC) screened a proportion of articles and discrepancies were discussed and resolved by the third reviewer.

##### Quality assessment

The Critical Appraisal Skills Programme (CASP) tool was used to assess the quality of the included studies as it was considered an appropriate tool for qualitative studies and is widely used (www.casp-uk.net). DC, LH and MD carried out critical appraisal and discussed any discrepancies of one point or more on the 10-point scale. The overall quality of the included studies was appraised as good, with scores ranging from 5.5 to 10. A summary of the quality appraisal is shown in Online Resource 4. No articles were excluded on the basis of poor quality. Findings were interpreted in the context of the quality markers and corresponding limitations of the included studies.

##### Data extraction

Data was extracted by DC and LH from included studies into a Microsoft Excel proforma, including: author; year of publication; country of study; study type; setting; relevant background and impetus for the study; methodological approach and specified methods; patient characteristics and demographics including cancer and comorbidity type; main findings relating to illness experience, psychosocial needs and supportive care; strengths and limitations and key relevant discussion points.

##### Data synthesis

Thematic synthesis, developed by Thomas and Harden, was chosen as an appropriate, transparent method for combining and interpreting qualitative findings [27, 28] (see protocol for full details on the chosen approach) for a heterogeneous body of evidence. This method allowed comparison of concepts and themes that relate different studies and uses a similar approach to developing descriptive themes and an interpretive account, as a grounded theory approach does with primary data analysis [31, 32].

##### Patient and public involvement

A consumer member of the research team (EB) was involved in the design and implementation of the systematic review, commenting and helping to shape the protocol and search strategy for the study, the synthesis and the paper drafting.

## Findings

**Fig. 1:**
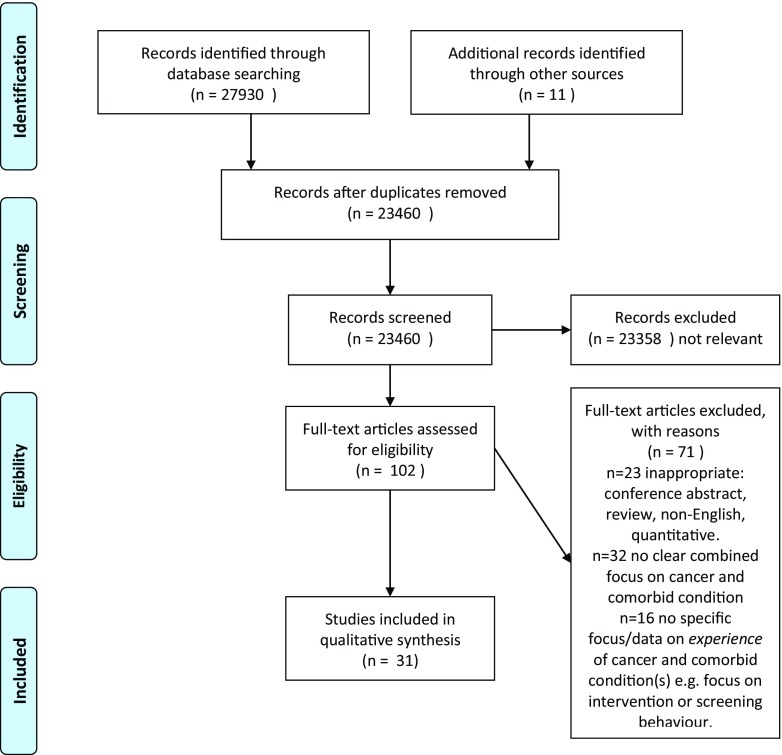
PRISMA flow diagram

Thirty-one articles were included in the final review [23, 33–62], based on 28 independent studies from an original yield of 27,941 papers identified through searching, reference snowballing, related articles and expert recommendation. The full screening process is outlined in the PRISMA flowchart in the PRISMA flow diagram (Fig. 1). The characteristics of the 31 included papers can be found in the characteristics of included studies (Table 1).

**Table 1:** Characteristics of included studies

Author (year)	Country of study/setting	Study type/method used	Study focus	Subjects/gender (*N*)	Age of subjects	Analysis/theoretical underpinnings reported	Cancer type (years since diagnosis)	Comorbid illness(es) reported	Themes evidenced in article
Baker [33] 2015	USA	QL/in-depth semi-structured interviews at two time points	Clinical care providers’ views on survivors’ weight management	Professionals: oncologists, surgeons, PCPs, nurses, dieticians. (*N* = 33)	?	Constant comparative analysis	Prostate, breast, non-Hodgkin’s lymphoma (years not reported)	Diabetes, hypertension	1, 2, 4, 5
Bartlett [34] 2012	UK	QL/in-depth interviews	Health professional views on needs assessment and communication with cancer patient with dementia	Healthcare professionals (*N* = 5)	?	Heidegger’s phenomenological approach	Not reported; professional interviews	Dementia	1, 2, 3, 4, 5
Beck [35] 2009	USA	Mixed/interviews and psychometric tests	Symptom experience, QoL and functional performance of cancer survivors at 1 and 3 months post-treatment	Male and female cancer patients (*N* = 52)	65+	Not reported	All—most common breast and prostate (1 and 3 years after diagnosis)	Arthritis, hypertension, diabetes, heart disease, lung disease, neuromuscular disease	1, 2, 3, 5
Clarke [36] 2013	Canada	QL/semi-structured interviews at two time points	Experience of multiple conditions in later life and influence of gender and age.	Male and female patients with multi-morbidity (*N* = 35)	73+	Patton’s thematic analysis	Not clearly reported (one bladder cancer)	Arthritis, back pain, heart disease, COPD	1, 2, 3, 4
Corner [37] 2013	UK	Mixed/QL free text comments analysis	How free text comments on PROMs added to understanding of QoL issues for survivors	Male and female cancer patients (*N* = 1056)	Over 16	Content analysis	Breast cancer, colorectal cancer, non-Hodgkin lymphoma, prostate cancer (1–5 years)	Hypertension, arthritis, osteoporosis, back pain	1, 2, 3, 4, 5
Courtier [38] 2016	UK	QL/case studies	Outpatient experiences of patients with dementia and oncology management	Male and female cancer/dementia patients and five HCPs (*N* = 16)	?	Wolcott’s framework for qualitative data analysis	Breast cancer, prostate cancer, head and neck cancers, pelvic cancer, colorectal (years not reported)	Dementia	1, 3, 4, 5
Dahlhaus [39] 2014	Germany	QL/semi-structured telephone interviews	German GPs’ views on involvement in patients’ cancer care	Male and female GPs (*N* = 30)	?	Mayring’s qualitative content analysis	Any professional interviews	Dementia, hypertension	5
Fenlon [40] 2013	USA	QL/in-depth semi-structured interviews	Lived experience, support and information needs of breast cancer patients with comorbid illness	Male and female patients (*N* = 48, 43 are male)	70–90 years	Braun and Clarke’s thematic analysis	Breast cancer (up to 10 years)	Cardiac conditions, arthritis, hypertension, diabetes	1, 2, 3, 5
Fix [41] 2014	USA	QL/longitudinal interviews	Hypertension self-management in patients with comorbidities	Male and female patients (*N* = 48, 43 are male)	Mean age 60 years	Grounded theory approach; Kleinman’s explanatory model as a framework	Prostate cancer, renal cancer (years not reported)	Hypertension, anxiety, arthritis, back pain, cardiovascular disease, COPD, depression, glaucoma, Parkinson’s disease	1, 2, 4, 5
Hannum [42] 2016	USA	QL/longitudinal interviews	Narratives of cancer among chronically ill older adults	Male and female patients (*N* = 16, 13 are female)	65 and over	Thematic analysis based on symbolic interactionism, constructivism and phenomenology	Breast, colon, brain, endometrial, oesophageal, GIST, large B cell lymphoma, pancreatic, stomach (within 1 year)	Diabetes, glaucoma	2, 3
Hershey [43] 2012	USA	Mixed/phone survey with two open-ended questions	Impact of cancer and its treatment on diabetes self-management	Male and female patients (*N* = 37)	50 and over	Qualitative content analysis	Breast, lung and pancreas (years not reported)	Diabetes (type I and II)	1, 2, 3, 4, 5
Kantsiper [44] 2009	USA	QL/focus group interviews	Needs and priorities of breast cancer patients, oncologists and PCPs.	Female patients; male and female professionals (*N* = 52)	?	Qualitative thematic analysis	Breast (1 year or more)	Diabetes, hypertension, renal issues	5
Loerzel [45] 2012	USA	QL/semi-structured interviews	Understanding post-treatment survivorship in older women with breast cancer	Female patients (*N* = 20)	65–86 years	Grounded theory approach and analysis	Breast (within 1 year)	Fibromyalgia, arthritis	1, 2, 3
Loerzel [46] 2013	USA	QL/semi-structured interviews	Understanding post-treatment survivorship in older women with breast cancer	Female patients (*N* = 20)	65–86 years	Grounded theory approach and analysis	Breast (within 1 year)	Fibromyalgia, arthritis	2, 3
Mason [23] 2014	UK	QL/in-depth serial interviews	Experience of advanced multi-morbidity and implications for palliative and end of life care	Male and female patients and carers (*N* = 37)	55–62 years (mean 76)	Thematic analysis; constructionist approach	Lung (years not reported)	Heart disease, respiratory, liver, renal failure, neurological conditions, dementia	2, 3, 4, 5
Morgan [47] 2015	UK	QL/in-depth serial interviews	Experience of advanced multi-morbidity and implications for palliative and end of life care	Male and female patients and carers (*N* = 37)	55–62 years (mean 76)	Thematic analysis; constructionist approach	Breast; professional interviews	Dementia and others non-specified	1, 5
Nanton [48] 2016	UK	QL/in-depth serial interviews	To examine the impact of uncertainty on identity in people with advanced multi-morbidity and the role of HCPs and health systems	Male and female patients and carers (*N* = 37)	41–92 years	Thematic analysis; based on social constructionism and ethnography	Lung, prostate (years not reported)	Stroke, ischaemic heart disease, dementia, osteoarthritis, hypertension	2, 3, 4, 5
Norman [49] 2001	Canada	QL/semi-structured interviews	Palliative care patients’ relationships with their family physicians	Male and female patients (*N* = 25)	28–84 years	Grounded theory approach	12 types of cancer (1 month-12 years)	Diabetes	5
Palmer [50] 2011	USA	Mixed/survey and interviews	Health-related goals of post-treatment colorectal cancer survivors	Male and female patients (*N* = 41)	33–87 years	Unclear; thematic analysis described	Colorectal (within 2 years)	Diabetes, chronic pain	2, 3, 4
Palmer [51] 2013	USA	QL/semi-structured interviews	Health-related goals of post-treatment colorectal cancer survivors	Male and female patients (*N* = 41)	33–87 years	Content analysis	Colorectal (within 2 years)	Diabetes, chronic pain	2, 4
Sada [52] 2011	USA	QL/semi-structured interviews	PCP and oncologists’ roles and communication in shared care of cancer patients	Male patients, male and female professionals (*N* = 24)	40–80+	Unclear; some form of thematic analysis	Colorectal (within 2 years)	COPD, cardiovascular disease, diabetes and others non-specified	1, 2, 3, 4, 5
Saunders-Sturm [31] 2003	USA	QL/semi-structured interviews	Lived experience of breast cancer	Female patients (*N* = 33)	47 years and over	Grounded theory approach; social constructionist	Breast (18 months-4 years)	Arthritis, asthma, chronic bronchitis, high blood pressure and diabetes	2, 3, 4
Sawin [54] 2012	USA	QL/semi-structured interviews	Ageing related experiences of women with breast cancer in unsupportive relationships	Female patients (*N* = 16)	50–84 years (mean 68.1)	Hermeneutic phenomenological analysis	Breast (1–31 years; mean 7.4)	Hypertension, chronic pain, COPD	1, 2, 3
Sinding [55] 2008	Canada	QL/semi-structured interviews at two time points	Older women’s experiences of cancer	Female patients (*N* = 15)	70 years and over	Grounded theory approach and analysis	Breast and gynaecological (within 3 years)	Diabetes, Chrohn’s disease, ‘mental illness’	1, 2, 3
Sowerbutts [56] 2015	UK	QL/in-depth interviews	Surgery decisions in older women with breast cancer	Female patients (*N* = 28)	70–99 years	Framework analysis	Breast (within 30 days)	Non-specified	1, 3, 5
Thomé [57] 2003	Sweden	QL/interviews	Experiences of older people living with cancer and its impact on daily life	Male and female patients (*N* = 64)	76–99 years (mean 87)	Latent content analysis	Breast, prostate, gastro, gynae, urogenital, skin, lung/larynx, haematological	Within 5 years	1, 2, 3, 4
Volker [58] 2013	USA	QL/focus group interviews	Cancer diagnosis for people with pre-existing functional limitations and topics for a wellness intervention programme	Female patients (*N* = 19)	21 and over (mean 59.5)	Patton’s qualitative content analysis	Breast, colorectal, bladder, gynae, melanoma, thyroid, kidney (10 years on average)	Arthritis, multiple sclerosis	1, 2, 3, 4, 5
Wallace [59] 2015	USA	QL/semi-structured interviews at two time points	Treatment experiences of newly diagnosed head and neck cancer patients and influence of age and time	Male and female patients (*N* = 41, 32 are male)	45–91 (mean 58.4)	Cresswell’s qualitative analysis; based on socio-emotional selective theory and Leventhal’s self-regulation model	Head and neck (within 2 weeks)	Heart disease, diabetes, Alzheimer’s, COPD, arthritis, fibromyalgia, liver failure	3, 4, 5
Wimberley [60] 2012	USA	QL/in-depth serial interviews	Influence of breast cancer on women’s meanings, understanding and self-identities over time.	Female patients (*N* = 15)	41–76 years (mean 59.4)	Hermeneutic analysis; phenomenological underpinnings	Breast (5–25 years)	Scleroderma and hypothyroidism	1, 2, 3, 4
Yoo [61] 2010	USA	QL/in-depth interviews	Psychosocial impact of breast cancer and social support for older women	Female patients (*N* = 47)	65–83 years	Grounded theory approach and analysis	Breast (within 4 years)	Heart disease and others non-specified	1, 2, 3, 4
Zhang [62] 2015	USA	Mixed/psychometric test and interviews	Universal and unique depressive symptoms in African American cancer patients	Male and female patients (*N* = 74)	Mean 62 years	Unclear; thematic analysis described	Not reported	Depression	2

The identified papers represented a heterogeneous range of studies including different cancer and comorbidity types. The majority of studies did not *focus* on the experience of cancer and comorbidity. It was often the case that comorbid conditions were mentioned briefly in relation to another issue, with only small amounts of qualitative data and limited discussion of specific issues relating to cancer and comorbidity. A small number of studies focused mainly on the experience of comorbidity or multi-morbidity and cancer was considered tangentially. Studies were largely exclusively qualitative, with five mixed methods papers, and include three PhD theses. Qualitative studies primarily used interviews (including longitudinal interviews), with a small number of focus group studies and one paper including case studies and observations. Two quantitative surveys included free-text comments.

The majority of studies were published from 2010 onwards, with only five included studies published prior to this. Papers were mostly published from high-income countries. The majority were US-based, followed by the UK, Germany, Sweden and Canada. Researchers came from a number of different disciplines, settings and health systems, all of which influence their approach, reporting style and thus the extent to which the results can be synthesised.

Studies were mostly set in secondary care hospitals, specialist clinics or specialist cancer centres; others included primary and community care settings (not all studies specified where participants were recruited from). The majority of studies included patient perspectives and two also included informal carer interviews. Six studies included health professional perspectives (two of these alongside patient interviews): three primary care and three specialist views.

Nine studies focused exclusively on breast cancer. Other common cancers included were prostate, lung, colorectal and lymphoma. Three studies did not specify a cancer type whereas others non-specifically reported on ‘any’ cancer type. Comorbid conditions were mostly reported in general but three studies focused on exclusive conditions: diabetes, dementia and depression. Common examples of other comorbid conditions featured were cardiac conditions including hypertension, arthritis, chronic pain, lung/respiratory conditions and stroke.

There was variation of stage and severity of cancer and/or comorbid condition, and in time since original diagnosis (depending on the aims and objectives of the included studies) such that experience could be expected to vary considerably depending on these characteristics, limiting opportunity for synthesis. It was important to consider these variations when interpreting differences in findings.

The total number of participants in a study ranged from five to 1056 (free-text comments from a survey of 3300 [37]. Eleven papers looked at women only; the rest included both men and women apart from one study with health professionals which did not specify gender. Experience of ageing in the context of illness was a key issue (mentioned in 11 papers), although interpretation of ‘older’ varied considerably from 50+ to 70+ with included participants in their late 90s so experiences were likely to vary extensively.

Studies varied considerably in their reporting of theoretical underpinnings and epistemology. Some described analysis without referring to a theoretical or methodological approach and only a few studies gave more detailed accounts of their theoretical stance and the methodological coherence.

### Synthesis

Five themes emerged from the analysis. The themes relating to each included study are listed in column 10 of Table 1.

### Interaction and impact of cancer and comorbidity

The interaction of having cancer and additional chronic illness reportedly produces a complex and increased burden of ill health for many,

‘They're morbidly obese, they have heart disease, diabetes, they all have orthopedic issues... breast cancer's just one thing’. (Nurse navigator, [33])

There is an indication in the literature reviewed that this complexity influences not only quality of life, and recovery (e.g. Corner et al. [37]), but also treatment decisions,

‘I could not have [general anaesthetic] because it affects my heart, you see’. (Patient 23, [56])

‘If you have got umm a person with reduced mental capacity [because of dementia] living on their own then the safety of giving chemotherapy is a risk’. (Oncologist, [38])

### Symptom experience

Related to *interaction and impact of cancer and comorbidity*, experiencing additional chronic ill health as well as cancer brought about a complex symptom burden that appeared to be mediated by stage and severity as well as proximity to diagnosis of the cancer and/or comorbidity. Evidence varied to suggest that some patients prioritised one condition over another; whereas, for others, they appeared to be entwined. Participants in some studies described cancer as merely a ‘bump in the road’; whereas, for others, the symptoms and effects were more enduring. For some, complexity of disease led to a blurring of symptoms and an inability to attribute symptomology to a particular condition; a source of fear of the cancer returning,

‘Dr C said that he doesn't really feel that I got too much to worry about, that they got it in time, but then how do they know if they got everything? ...And when my lungs are bad [asthma], it frightens you’. (Judith, [55])

### Illness expectations and identity

Ability to adjust and cope with a complex burden of ill health was influenced by past experience of illness (including sequencing of conditions, i.e. whether the cancer or the comorbid condition came first) as well as notions of ageing and expectations of ailing health and functional ability. Expecting illness with advancing age helped to ‘lessen the shock’ of cancer diagnosis for some participants in the included studies [45], captured by those participating in Mason et al as ‘old not ill’ [23].

Maintaining a sense of control and one’s existing personal identity appeared to be an important part of illness experience for participants in many included studies,

‘One feels sorry for oneself, in the sense that one loses one's independence and you become dependent on others’. (91 year old patient with back problems, cancer, heart disease and other conditions, [36])

### Managing medications and self-management

A smaller number of studies referred to managing medications as being a challenge for people living with cancer and other chronic illness at the same time. In addition to issues such as being refused trial participation due to contraindicated medications and the financial cost of medications in relevant health systems, one of the concerns raised was the ability to self-manage the range of medications needed. Patients also had to look out for contraindications as the communication between primary and secondary care or between different specialists was not always optimal, meaning the patient often had to champion their position,

‘The fact that I'm on this pain control regimen, doctors wanted to ignore it, he didn't want to deal with someone on Fentnyl patch and Oxycodone, just didn't want to deal with that. So if you don't advocate for yourself, forget it...sometimes you get tired of fighting for yourself and trying to educate everybody’. (Cancer patient, [58])

In terms of self-management, studies emphasised the need for shared care or supported self-management (including looking after overall health and well-being), and the need for resources to enable this and to take the pressure off primary care in providing follow-up care and support for this patient group.

### Role of primary and secondary care

The evidence suggests that oncologists do not often see the management of comorbid conditions as being part of their role or within their perceived level of competency. GPs were more likely to regard holistic condition management as part of their role and advocated a patient-centred ‘joined up’ approach. For example, a medical oncologist interviewed by Sada et al. said:

‘I encourage patients to continue the management of their [chronic illness] with their PCP...I'm so subspecialised that I don't really feel comfortable managing [it]’. (Medical oncologist 6, [52])

However, primary care practitioners did not always feel comfortable managing advanced cancer symptoms, potentially leading to a fragmented experience of care, as another study suggests:

‘[The FP (family physician) is] still looking after their diabetes…One kind of doctor does this; he does that: [to] each their own. Symptom control is not in their hands…You leave that to the specialists. She’s just a family physician’. (Cancer patient, [49]).

Managing cancer multi-morbidity in primary care carries many challenges:

‘…Actually most of our patients have five or six issues, and so we’re trying to manage their diabetes or hypertension or renal insufficiency and then end up with a breast issue, and it’s impossible…that’s like more than an hour’s worth of time where you have like four patients scheduled in 15-min increments, and it’s overwhelming’. (Primary Care Professional, [44])

## Discussion

### Summary

**Fig. 2:**
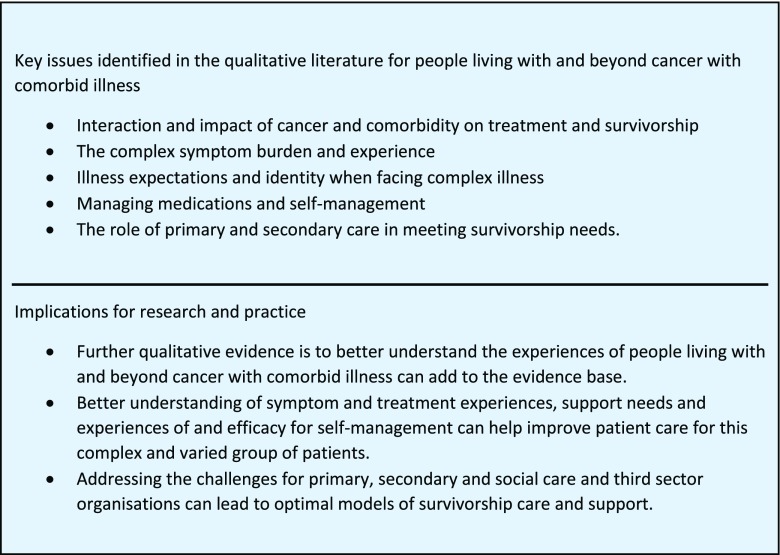
Identified issues and implications for research and practice

This systematic review of the evidence relating to the experience of living with and beyond cancer with comorbid illness identified a small number of heterogeneous papers with a range of aims and perspectives. Qualitative data on this specific topic was difficult to identify as comorbidities were often mentioned in passing or ‘buried’ in articles with a different dominant focus. From the 31 included studies, a number of themes emerged to give a summary of the current evidence and help identify topics for further investigation (see Fig. 2). These issues relate to the burden of symptoms, the combined impact on one’s illness experience and how adjustment was entwined with previous experience of illness, illness expectations and the severity of the illness burden. Dealing with polypharmacy, self-managing and the related challenges and demand for health services, including the relationship between primary and specialist care, were also identified as important concerns. Overall, there is need to grow the evidence base on this topic, in order to address the support needs of the growing number of cancer survivors living with other chronic conditions, their informal carers and health care professionals.

### Place in the wider literature

The findings from the review sit within a wider literature examining experiences of cancer survivors and the role for primary and secondary care, as well as literature around multi-morbidity, e.g. [20, 63–68]. There have been a number of intervention studies to support patients living with and beyond cancer [69, 70], and to consider the views of health care providers [21, 71]. There is also a recognition that guidance is needed for a model of survivorship care that acknowledges the challenges for and complexity of this patient group, including measuring comorbid conditions and managing them in the treatment and survivorship contexts [12, 13, 72].

A number of potential theoretical positions may provide insight to the emergent themes from the synthesis. Indeed, a small number of included studies refer to theory in order to make sense of their findings. For example, issues of symptom burden and people’s illness expectations and identity relate to experience of illness theory around keeping illness at the boundaries of one’s self concept in an attempt to maintain one’s existing personal identity (Charmaz 2000) and can be understood in the context of biographical disruption, flow and continuity [73, 74] and Frank’s illness narratives [75]. Further in-depth research can revisit the potential contribution of these theoretical insights to the experience of cancer and comorbid illness.

The issue of the pressure on primary care to manage cancer survivor follow-up and for patients to self-manage is one that has been problematised in the sociological literature, marking a shift toward ‘living with and beyond cancer’. It is argued that the term ‘survivor’ is heavily loaded with expectations of optimism in ‘fighting’ the cancer battle with an emphasis on individual responsibility and accountability for lifestyle change and secondary prevention [76, 77], with implications for the ‘burden of treatment’ work expected of patients and their families, and with repercussions for health services [78].

### Strengths and limitations

This review brings together studies exploring experience of illness. It focuses on qualitative findings to meet the aims of identifying contextual evidence of psychosocial issues and supportive care needs.

The articles included in the review come from differing settings and foci. Due to the broad exploratory nature of the topic, it was difficult to design a search strategy that was sensitive and specific. Given the ‘snippets’ of qualitative data relating to the topic of cancer and comorbid illness, there may be additional articles containing relevant data that were not identified. Additional methods of snowball referencing and related articles searching as well as expert consultation were undertaken to address this potential limitation. Also, despite the heterogeneity of the studies, it was possible to deduce some common themes, suggesting that a diverse sample of papers from a number of different settings can enable a level of conceptual saturation of ideas [79], and remains true to the aim of producing an abstract synthesis that is ‘faithful to the group of studies from which it was extracted’ [80].

### The research agenda

While multi-morbidity in cancer survivors is often highlighted as a key issue in literature and policy, qualitative research to date has yet to focus on it as a substantive concern. Reviewing the qualitative evidence on this topic has identified key themes that warrant further exploration and focus. Variations in symptoms burden and the interaction and impact on one’s illness experience have been indicated in the included studies. Further, qualitative work is needed to explore the influence of stage of survivorship, severity of the concomitant illnesses and the meaning attributed to cancer in relation to other chronic conditions. The challenges for health services have been identified in the literature: there is a call for further work to better understand the difficulties facing primary and secondary care in managing survivorship care in the context of multi-morbidity and what patients want from services in this context. Finally, carers’ views have been largely under-represented and it is important to explore the additional strain that caring for someone with cancer and additional chronic conditions imposes. These issues have also emerged from research prioritisation work carried out by the authors in relation to this topic (in draft), as well as the wider context of the recent National Cancer Research Institute Top 10 research priorities for living with and beyond cancer (www.NCRI.org.uk/lwbc), and have combined to inform the design (including aims, sampling decisions and interview topic guide) of an in-depth, multi-perspective interview study with patients, carers and health care professionals.

## Conclusions

Further, qualitative evidence is required to understand better symptom experience, stage, severity and proximity to diagnosis of disease, views on and experiences of current care, support for self-management and the role of primary and secondary care, as well as carer experiences, to develop optimal care for the complex needs of this growing and diverse group of cancer survivors.

## Electronic supplementary material

ESM 1 (PDF 337 kb)

ESM 2 (PDF 486 kb)

ESM 3 (PDF 449 kb)

ESM 4 (PDF 402 kb)
