# Supplementary material for: Living with and beyond cancer with comorbid illness: a qualitative systematic review and evidence synthesis
Source: J Cancer Surviv. 2019 Jan 26;13(1):148–59. doi: 10.1007/s11764-019-0734-z (PMC6394454; doi:10.1007/s11764-019-0734-z)
Supplement: Supplementary file 1 — (PDF 337 kb) [file 11764_2019_734_MOESM1_ESM.pdf]

Living with and beyond cancer with comorbid illness: a qualitative systematic review and evidence synthesis, Journal of Cancer Survivorship, Debbie Cavers, Liset Habets, Sarah Cunningham-Burley, Eila Watson, Elspeth Banks, Christine Campbell

Corresponding author: Debbie Cavers, University of Edinburgh, Scotland, UK, Debbie.Cavers@ed.ac.uk

Online Resource 1: List of Comorbid Conditions

Hypertension
Depression
Painful condition
Asthma
Coronary Heart Disease
Dyspepsia
Diabetes
Thyroid Disorders
Rheumatoid arthritis, other inflammatory
polyarthropathies and systemic connective tissue disorders
Hearing loss
Chronic Obstructive Pulmonary Disease
Anxiety and other neurotic, stress-related and somatoform disorders
Irritable Bowel Syndrome
Cancer
Alcohol Problems
Other psychoactive substance misuse
Constipation
Stroke or transient ischaemic attack
Chronic kidney disease
Diverticular disease of intestine
Atrial fibrillation
Peripheral vascular disease
Heart failure
Prostate disorders
Glaucoma
Epilepsy
Dementia
Schizophrenia
Psoriasis or eczema
Inflammatory bowel disease
Migraine
Blindness and low vision
Chronic sinusitis
Learning disability
Anorexia or bulimia
Bronchiectasis
Parkinson’s disease
Multiple Sclerosis
Viral hepatitis
Chronic liver disease

Source: Adapted from: Barnett K, Mercer SW, Norbury M, Watt G, Wyke S, Guthrie B. Epidemiology of multimorbidity and implications for health care, research, and medical education: a cross-sectional study. *Lancet*, 2012;380(9836):37-43.
