# Supplementary material for: Living with and beyond cancer with comorbid illness: a qualitative systematic review and evidence synthesis
Source: J Cancer Surviv. 2019 Jan 26;13(1):148–59. doi: 10.1007/s11764-019-0734-z (PMC6394454; doi:10.1007/s11764-019-0734-z)
Supplement: Supplementary file 2 — (PDF 486 kb) [file 11764_2019_734_MOESM2_ESM.pdf]

Living with and beyond cancer with comorbid illness: a qualitative systematic review and evidence synthesis, Journal of Cancer Survivorship, Debbie Cavers, Liset Habets, Sarah Cunningham-Burley, Eila Watson, Elspeth Banks, Christine Campbell

Corresponding author: Debbie Cavers, University of Edinburgh, Scotland, UK, Debbie.Cavers@ed.ac.uk

Online Resource 2: Inclusion and exclusion criteria

Inclusion criteria:

- Study population – Adults (over 18) with a diagnosis of cancer and at least one other condition (as specified in appendix 1), carers and health professionals
- Articles relating to experience of illness from patient, carer and professional perspectives
- Articles focusing on areas denoted in the dimensions of interest
- Studies with a qualitative empirical design
- Articles published in English

Exclusion criteria:

- Articles not meeting the above inclusion criterion.
- Articles published before 2000
- Articles focusing on long term side effects of cancer treatment or second primary cancers alone.
