# Supplementary material for: Living with and beyond cancer with comorbid illness: a qualitative systematic review and evidence synthesis
Source: J Cancer Surviv. 2019 Jan 26;13(1):148–59. doi: 10.1007/s11764-019-0734-z (PMC6394454; doi:10.1007/s11764-019-0734-z)
Supplement: Supplementary file 3 — (PDF 449 kb) [file 11764_2019_734_MOESM3_ESM.pdf]

Living with and beyond cancer with comorbid illness: a qualitative systematic review and evidence synthesis, Journal of Cancer Survivorship, Debbie Cavers, Liset Habets, Sarah Cunningham-Burley, Eila Watson, Elspeth Banks, Christine Campbell

Corresponding author: Debbie Cavers, University of Edinburgh, Scotland, UK, Debbie.Cavers@ed.ac.uk

Online Resource 3: Example search strategy for Medline

1. Comorbidity/

2. (Multimorbid$ or multi-morbid$ or Co-morbid$).mp. [mp=title, abstract, original title, name of substance word, subject heading word, keyword heading word, protocol supplementary concept word, rare disease supplementary concept word, unique identifier]

3. Chronic Disease/ or (Chronic adj5 (illness$ or condition$ or disease)).tw.

4. (Long term adj5 (condition$ or illness$ or disease$)).mp. [mp=title, abstract, original title, name of substance word, subject heading word, keyword heading word, protocol supplementary concept word, rare disease supplementary concept word, unique identifier]

5. 1 or 2 or 3 or 4

6. Neoplasms/ or Cancer.mp or (Tumour or tumor or Oncology or Neoplasm).mp. [mp=title, abstract, original title, name of substance word, subject heading word, keyword heading word, protocol supplementary concept word, rare disease supplementary concept word, unique identifier]

7. Qualitative Research/ or Qualitative.mp or Interview/ r Interview$.mp or In-depth.mp

8. (Focus Group or or Ethnograph$ or Observation$ or Participant$ or Respondent$ or View$ or Belief$ or Attitude$ or Awareness or Perspective$ or Understanding$ or Findings or Grounded Theory or Social Construction$ or Theoretical or Phenomenolog$).mp. [mp=title, abstract, original title, name of substance word, subject heading word, keyword heading word, protocol supplementary concept word, rare disease supplementary concept word, unique identifier]

9. 7 or 8

10. Psychosocial.mp. or Stress, Psychological/ or Social Support/ or Psycho-social.mp or “Quality of Life”/ or “Experience of Illness”.mp or Experience adj5 Illness or Attitude to Health/ or “Quality of Life”.mp or Happ$.mp or Emotion$.mp. or Emotions/

11. Patient satisfaction.mp. or Patient Satisfaction/ or “Quality of Health Care”/ or “Patient Experience”.mp or Satisfaction.mp or “Information Preferences”.mp or Decision Making/ or (Decision-making or Decision Making).mp or (Informed choice or Informed Decision Making).mp or Self management.mp. or Self Care/ or "Continuity of Patient Care"/ or Integrated care.mp

12. Access to Health Care.mp. or Health Services Accessibility/

13. Identity.mp. or gender.mp. or Gender Identity/

14. (Health behaviour or Health behavior).mp.

15. (Help-seeking or Help seeking).mp.

16. (Well-being or Well being).mp. or Holistic.mp or Continuity of Care.mp or “Continuity of Patient Care”/ or (Fragmented adj5 care).mp or (joined-up or Joined Up).mp or Integrated care.mp or Diversity of Care.mp.

17. Primary care.mp. or Primary Health Care/ or Secondary care.mp. or Secondary Care/ or Community Care.mp or Community Health Services/ or Ambulatory Care.mp. or Ambulatory Care/ or "Delivery of Health Care, Integrated"/ or Preventive Health Services/ or Family Practice/ or Shared care.mp or Collaborat$ care.mp

18. (Patient-centred care or Patient centred care or Patient-centred or Person-centred or Patient centred or Person centred).mp or Patient-Centered Care/

19. Palliative Care/ or Supportive care.mp. or palliative.mp.

20. "Health Services Needs and Demand"/ or unmet need.mp. or Health Services Research/ or Depriv$.mp. or (Socio-economic or Socioeconomic).mp. or Information needs.mp.

21. Survivor$.mp. or Survivors/

22. Patient Care Planning/ or Care plan.mp. or (Follow-up or Follow up).mp.

23. ("use of service$" or "service use" or "service adj5 use").mp.

24. 10 or 11 or 12 or 13 or 14 or 15 or 16 or 17 or 18 or 19 or 20 or 21 or 22 or 23

25. 5 AND 6 AND 9 AND 24
