# Supplementary material for: Living with and beyond cancer with comorbid illness: a qualitative systematic review and evidence synthesis
Source: J Cancer Surviv. 2019 Jan 26;13(1):148–59. doi: 10.1007/s11764-019-0734-z (PMC6394454; doi:10.1007/s11764-019-0734-z)
Supplement: Supplementary file 4 — (PDF 402 kb) [file 11764_2019_734_MOESM4_ESM.pdf]

Living with and beyond cancer with comorbid illness: a qualitative systematic review and evidence synthesis, Journal of Cancer Survivorship, Debbie Cavers, Liset Habets, Sarah Cunningham-Burley, Eila Watson, Elspeth Banks, Christine Campbell

Corresponding author: Debbie Cavers, University of Edinburgh, Scotland, UK, Debbie.Cavers@ed.ac.uk

Online Resource 4: Critical appraisal of included studies using the Critical Appraisal Skills Programme (CASP) tool

Author	Year	Total score (max 10)	1. Clear statement of the aims?	2. Is qualitative methodology appropriate?	3. Research design appropriate?	4. Recruitment strategy appropriate?	5. Data collection that addressed research issue?	6. Relationship between researcher and participants?	7. Ethical issues considered?	8. Data analysis sufficient?	9. Clear statement of findings?	10. How valuable is the research?
1.Baker	2015	10	Y	Y	Y	Y	Y, no 5 or 7	Y, no 2	Y, no 2	Y	Y	Y
2.Bartlett	2012	5.5/ 6	Y	Y	Y	N	Y, no 1, 5 or 7	N	N	N	Y, no 3 or 4	Y
3.Beck	2009	9	Y	Y	Y, no 1 (don't justify a mixed methods approach)	Y, no detail on 1, no 2 or 3	Y, no 3 or 7, 5 n/a	N, no 1 or 2	Y, no 2	Y, no 5 or 6	Y, no 3	Y
4.Clarke	2014	9	Y	Y	Y	Y	Y	Y	Y	Y	Y	N, not very clear
5.Corner	2013	9	Y	Y	Y	Y, no 3	Y, no 5 or 7	N, no 1 or 2	Y, no 2	Y, no 5 or 6	Y, no 2	Y
6.Courtier	2015	9	Y	Y	Y	Y, no 2	Y, no 4 or 7	N	Y	Y, no 5 or 6	Y, no 3 and more detail on 2 would be good	Y
7.Dahlhaus	2014	9	Y	Y	Y	Y	Y, no 5	N, no 1 or 2	Y	Y, no 3 or 6	Y	Y, more detail on 2 needed
8.Fenlon	2012	9	Y	Y	Y	Y	Y	N, no 1 or 2	Y	Y	Y	Y
9.Fix	2014	9	Y	Y	Y	Y, no detail on 2	Y, no 4, 5 or 6	N	Y, little detail on 2	Y, no 5 or 6. I am not sure about	Y	Y

										a grounded theory approach when they are using a particular framework 'the Explanatory Mdoel'		
10.Hannum	2015	9	Y	Y	Y	Y, no 1	Y, no 5 or 7	N, no 1 or 2	Y	Y, no 5 or 6	Y	Y
11.Hershey	2012	8	Y	Y	Y	Y	Y, no 5 or 7	N	Y, no 2	Y, more 4 needed, no 6	Y, no 3	Y
12.Kantsiper	2009	8.5	Y, a little bit vague	Y	Y, some sensitive personal issues may have been missed from patient groups	Y, potential missing voices as recruitment was through support groups.	Y	N	Y, limited report	Y, large volume of data so could have presented more	Y	Y
13.Loerzel	2012	9	Y	Y	Y	Y	Y	N	Y	Y	Y	Y
14.Loerzel	2013	9	Y	Y	Y	Y, no 3	Y	N	Y, no 3	Y, no 6	Y	Y
15.Mason	2014		Y, no 1	Y	Y	Y, no 2 or 3	Y, no 3 or 7	N	Y, no 2	Y, no 5 or 6	Y	Y
16.Morgan	2015	7.5	Y	Y	Y	Y	Y	N	Y	N	Y, limited	Y
17.Nanton	2016	9.5	Y	Y	Y	Y	Y	Y	Y	Y	Y	Y
18.Norman	2001	7	Y	Y	N, Unsure about content analysis with a grounded theory approach	N, convenience sample in one location	Y	N	Y	Y	Y	Y
19.Palmer	2011	7.5	Y	Y	Y	Y, no 2 or 3	Y, no 3 or 7	N	Y, no 2	Y, no 3 or 6	Y	Y, no 3

20.Palmer	2013	7.5	Y	Y	N	Y, no 2 or 3	Y, no 3 or 7	N	Y, no 2	Y, no 3 or 6	Y	Y, no 3
21.Sada	2017	8	Y	Y	Y	Y, no 3	Y	N	Y, not enough on 2	Can't tell, no 1, 2, 5 or 6	Y	Y
22.Saunders Sturm	2003	10	Y	Y	Y	Y	Y	Y	Y	Y	Y	Y
23.Sawin	2012	8	Y, no explicit 1 in main paper	Y	Y	Y, no 3	Y, no 3 or 7	N	N, not enough given the highly sensitive nature of the topic.	Y, no 6	Y	Y
24.Sinding	2008	10	Y	Y	Y	Y	Y	Y	Y	Y	Y	Y
25.Sowerbutts	2015	8	Y	y	Y	Y, no 2 or 3	Y	N	Y, no 2	Y, no 6	Y	Y
26.Thome'	2013	9	Y	Y	Y	Y, no 2	Y	N	Y	Y, no 6	Y	Y
27.Volker	2013	8	Y	Y	Y	Y, no 2 and limited 3	Y, no 6	N	Y, no 3 evident?	Y, no 6	Y, no 3	Y
28.Wallace	2015	9	Y	Y	Y	Y	Y, no 5 or 7	Y, not explicitly discussing role and impact	Y	Y	Y	Y
29.Wimberley	2012	9.5	Y	Y	Y	Y	Y, no 7	Y	Y	Y	Y	Y
30.Yoo	2010	6.5	Y	Y	Y	Not clear	Y, no 5 or 7	N	N	Y	Y	Y
31.Zhang	2015	8	Y	Y	Y	Y	Y, no 7	Mention of race of researcher but not why it is significant	N	Y, no 5 or 6	Y	Y
